# Beyond CAR-T: Preventing and Treating Relapse in B-Cell Haematological Malignancies

**DOI:** 10.3390/cells15181660

**Published:** 2026-09-14

**Authors:** Martina Canichella, Elisabetta Abruzzese, Mariagiovanna Cefalo, Valentina Gianfelici, Carla Mazzone, Gentiana Elena Trotta, Roberta Laureana, Luca Cupelli, Maria Ilaria Del Principe

**Affiliations:** 1 Division of Hematology, Ospedale S. Eugenio, Piazzale dell’Umanesimo 10, 00144 Rome, Italy; elisabetta.abruzzese@uniroma2.it (E.A.); mariagiovanna.cefalo@aslroma2.it (M.C.); valentina.gianfelici@aslroma2.it (V.G.); carla.mazzone@aslroma2.it (C.M.); gentianaelena.trotta@aslroma2.it (G.E.T.); roberta.laureana@aslroma2.it (R.L.); luca.cupelli@aslroma2.it (L.C.); mariailaria.delprincipe@aslroma2.it (M.I.D.P.); 2 Department of Biomedicina e Prevenzione, Tor Vergata University, 00133 Rome, Italy

**Keywords:** CAR-T cell therapy, acute lymphoblastic leukemia, large B-cell lymphoma, multiple myeloma, post-CAR-T relapse, consolidation therapy, allogeneic stem cell transplantation

## Abstract

Chimeric antigen receptor (CAR) T-cell therapy has revolutionized the treatment landscape of relapsed/refractory (R/R) B-cell acute lymphoblastic leukemia (B-ALL), non-Hodgkin lymphoma (NHL), and multiple myeloma (MM); however, a substantial proportion of patients eventually experience disease relapse despite an initial response. This review provides a brief overview of CAR-T cell therapy, including its manufacturing process and associated toxicities, before examining the biological mechanisms underlying post-CAR-T relapse in B-ALL, NHL—particularly large B-cell lymphoma (LBCL)—and MM. We critically review current and emerging strategies to prevent and manage relapse. Strategies for relapse prevention primarily include consolidation approaches, such as allogeneic hematopoietic stem cell transplantation (allo-HSCT), maintenance with targeted agents (e.g., tyrosine kinase inhibitors in Philadelphia chromosome-positive B-ALL), and CAR-T cell reinfusion, whereas relapse management is disease-specific and increasingly incorporates novel therapeutic agents, including bispecific antibodies (BsAbs) and other targeted therapies. However, current evidence remains largely preliminary and is limited by the paucity of randomized prospective trials.

## 1. Introduction

Chimeric antigen receptor (CAR) T-cell therapy has revolutionized the treatment of relapsed/refractory (R/R) B-cell hematological malignancies and is now established as a standard therapeutic option for patients with B-cell acute lymphoblastic leukemia (B-ALL), non-Hodgkin lymphoma (NHL), and multiple myeloma (MM) [1,2,3,4,5,6,7,8,9,10,11]. Since the first regulatory approval of tisagenlecleucel (tisa-cel) by the U.S. Food and Drug Administration (FDA) and the European Medicines Agency (EMA) in 2017 for children and young adults with R/R B-ALL, several additional CD19- and B-cell maturation antigen (BCMA)-directed CAR T-cell products have been introduced into clinical practice, substantially expanding the therapeutic armamentarium for B-cell malignancies. Currently, the approved CD19-directed CAR T-cell products include tisa-cel, axicabtagene ciloleucel (axi-cel), brexucabtagene autoleucel (brexu-cel), and lisocabtagene maraleucel (liso-cel). For BCMA-directed CAR T-cell therapy, the currently approved products are idecabtagene vicleucel (ide-cel) and ciltacabtagene autoleucel (cilta-cel) (Table 1) [1,2,3,4,5,6,7,8,9,10,11]. Importantly, increasing clinical experience, together with standardized toxicity grading systems and improved supportive care strategies, has substantially improved the safety profile of CAR T-cell therapy [12]. Although cytokine release syndrome (CRS) and immune effector cell-associated neurotoxicity syndrome (ICANS) remain the hallmark toxicities associated with CAR T-cell therapy, both complications are now largely predictable and manageable in experienced centers, allowing the broader implementation of this therapeutic approach in routine clinical practice [13,14,15].

Despite the unprecedented efficacy of CAR T-cell therapy and compelling long-term evidence supporting its curative potential in a subset of patients, durable disease control is not achieved in a proportion of individuals. Relapse therefore remains the principal cause of treatment failure and one of the greatest challenges in contemporary cellular immunotherapy. In B-ALL, approximately 40–60% of patients who achieve complete remission (CR) ultimately relapse, most frequently within the first two years after infusion [16]. In large B-cell lymphoma (LBCL), durable remissions are achieved in 35–45% of treated patients, while nearly 50–60% eventually experience disease progression or relapse, with the majority of events occurring during the first 24 months after infusion [17,18]. Conversely, patients who remain in remission beyond this landmark have a high probability of sustained long-term remission, supporting the existence of a plateau in progression-free survival (PFS) and the curative potential of CAR T-cell therapy in a subset of patients [19].

Similarly, BCMA-directed CAR T-cell therapy has dramatically improved outcomes in R/R MM, producing overall response rates (ORRs) exceeding 80%. However, despite these remarkable results, disease progression remains the major limitation of this therapeutic approach [8,10]. In the pivotal registration studies, median PFS reached 8.8 months after ide-cel and 34.9 months after cilta-cel, indicating that relapse represents the principal barrier to durable disease control even after highly effective BCMA-directed CAR T-cell therapy [20]. Consequently, improving the durability of CAR T-cell responses and developing effective strategies to prevent or overcome post-CAR-T relapse have become major priorities in both clinical practice and translational research [21].

The post-CAR-T setting encompasses two major clinical scenarios: consolidation of remission and treatment of relapse. Consolidation strategies aim to reduce the risk of disease recurrence in patients achieving CR and are generally tailored according to disease-specific and patient-specific relapse risk. These approaches may include allogeneic hematopoietic stem cell transplantation (allo-HSCT), maintenance therapy with targeted agents or immunomodulatory drugs, and, in selected cases, repeat CAR T-cell infusion. Conversely, the management of relapse requires a comprehensive understanding of the biological mechanisms responsible for treatment failure, including limited CAR T-cell expansion or persistence, functional T-cell exhaustion, antigen modulation or loss, lineage switch, and the establishment of an immunosuppressive tumor microenvironment [22]. As the therapeutic armamentarium continues to expand with bispecific antibodies (BsAbs), dual-target CAR T cells, next-generation engineered cellular products, and novel antigen-directed therapies, reassessment of tumor antigen expression and characterization of the mechanisms underlying immune escape have become essential for selecting the most appropriate salvage strategy.

In this rapidly evolving therapeutic landscape, this review aims to (i) briefly summarize CAR T-cell structure and manufacturing processes; (ii) discuss the biological mechanisms underlying post-CAR-T relapse in B-ALL, LBCL, and MM; and (iii) critically review current and emerging strategies for relapse prevention and treatment. To identify relevant evidence for this review, a literature search was conducted using PubMed/MEDLINE and Web of Science, focusing on studies addressing CAR-T cell therapy, mechanisms of treatment failure and relapse, and strategies to overcome resistance. Clinical trials, observational and translational studies, and relevant review articles were considered. Additional references were identified through screening the reference lists of the selected publications.

## 2. CAR-T Structure, Manufacturing, and Clinical Application in Hematologic Malignancies

T cells are central effectors of adaptive immunity, naturally detecting and responding to antigens through the T cell receptor (TCR), a transmembrane heterodimer that recognizes peptides presented in the context of major histocompatibility complex (MHC) molecules. This physiological recognition mechanism, however, is intrinsically limited by MHC restriction and by tumor-associated immune evasion strategies, which can blunt the efficacy of endogenous antitumor immunity [23]. CAR-T cell therapy was developed to overcome these constraints, redirecting T cell cytotoxicity toward MHC-independent tumor-associated antigens. This engineering strategy has translated into unprecedented clinical outcomes in R/R hematologic malignancies, including B-ALL, NHL, and MM, and has since become an integral component of the therapeutic armamentarium in modern hematology. The present paragraph provides an overview of CAR construct architecture and the manufacturing process underlying autologous CAR-T products. Table 1 summarizes the CAR T-cell products, pivotal clinical trials, efficacy, safety, and long-term outcomes.

### 2.1. CAR-T Cell Structure

**Figure 1:**
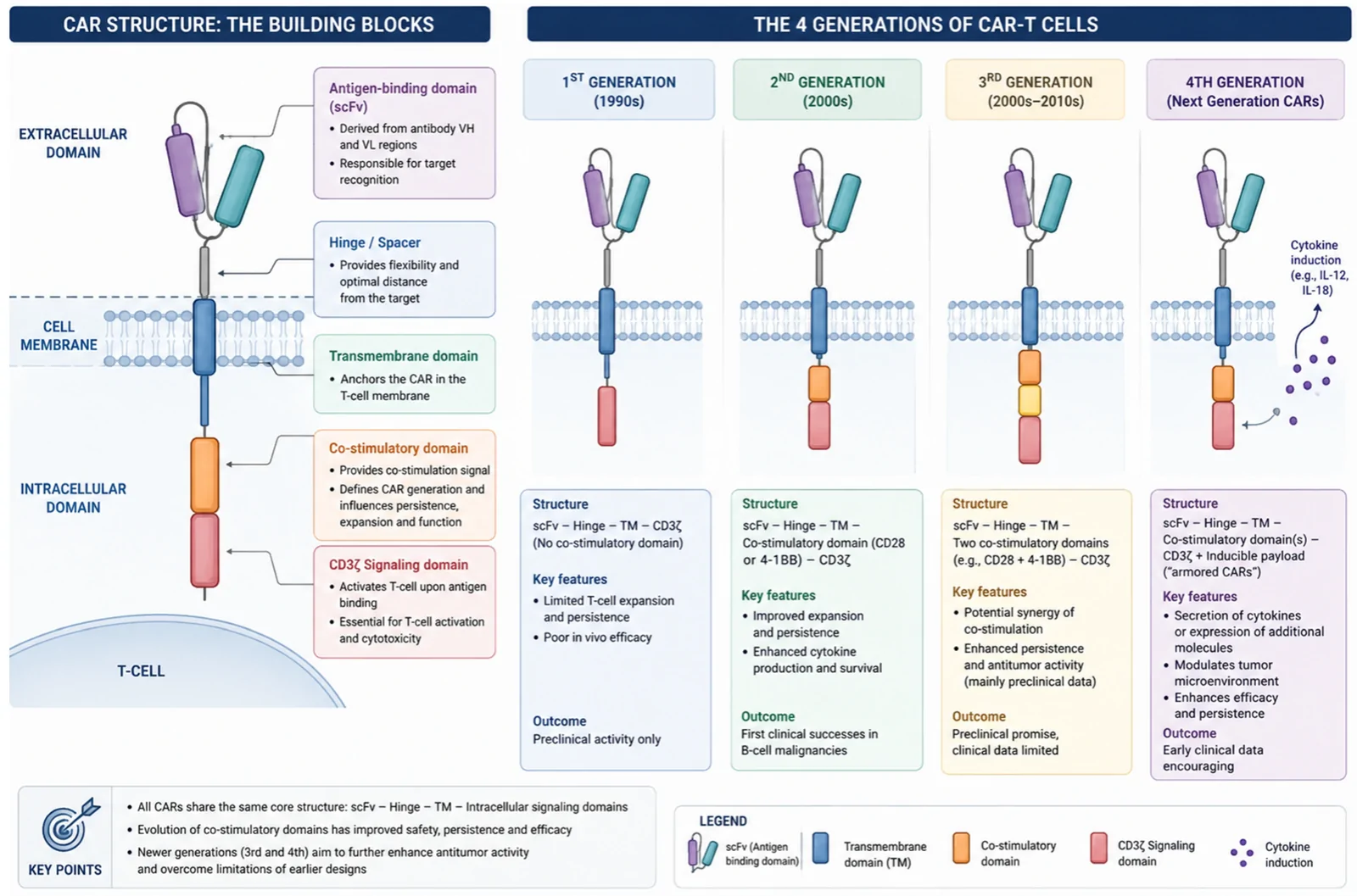
CAR construct and different CAR-T generation.

CARs are synthetic receptors composed of four main structural components: an extracellular antigen-binding domain, typically a single-chain variable fragment (scFv) derived from an antibody; a spacer (or hinge) region; a transmembrane domain anchoring the receptor to the T-cell membrane; and one or more intracellular signaling domains responsible for T-cell activation (Figure 1). Unlike the T-cell receptor (TCR), the scFv binds to surface antigens directly, without MHC restriction.

Based on the composition of the intracellular signaling domains, CARs are classified into five generations. First-generation CARs included only the CD3ζ chain [22,23]. Second- and third-generation CARs added one and two co-stimulatory domains, respectively, more closely reproducing physiological T-cell signaling and improving T-cell expansion and persistence [24,25]. All currently approved products are built on a second-generation design, incorporating either 4-1BB or CD28 as the co-stimulatory domain. Fourth-generation CARs, or T cells Redirected for Universal Cytokine-mediated Killing (TRUCKs), further incorporate inducible cytokine expression or other immunomodulatory elements to modulate the tumor microenvironment, an approach particularly relevant in solid tumors [26]. More recently, fifth-generation CARs have been developed by incorporating a truncated intracellular IL-2 receptor β-chain (IL-2Rβ) domain, which provides a STAT3 binding site, between the CD3ζ and co-stimulatory domains of a second-generation backbone; this enables antigen-dependent JAK-STAT signaling alongside canonical activation, promoting proliferation, reduced terminal differentiation, and improved persistence in preclinical models [27]. Fifth-generation constructs remain at an earlier stage of clinical development compared with second-generation products, and their long-term safety and efficacy in humans have yet to be established.

### 2.2. CAR-T Cell Manufacturing Process

CAR-T cells used for clinical applications are classified as advanced therapy medicinal products (ATMPs), and their manufacture is regulated by specific quality and safety requirements established by regulatory agencies, including the FDA and EMA. CAR-T cell therapy is a multistep process in which patients’ autologous T cells are collected, genetically engineered to express a synthetic antigen receptor, expanded ex vivo, and subsequently reinfused to mediate targeted antitumor activity. The manufacturing process consists of five sequential steps:

#### 2.2.1. Leukapheresis and T-Cell Collection

Peripheral blood mononuclear cells are collected through leukapheresis, from which T cells are subsequently enriched. Leukapheresis products may be cryopreserved; however, fresh leukapheresis material is increasingly preferred in several manufacturing settings to reduce processing delays and shorten overall manufacturing time.

#### 2.2.2. T-Cell Activation and Genetic Engineering

At the manufacturing facility, T cells are activated through CD3/CD28 stimulation in the presence of cytokines (e.g., IL-2, IL-7, and/or IL-15), promoting cell-cycle entry and increasing susceptibility to gene transfer [28]. The CAR transgene is subsequently introduced using viral vectors, including γ-retroviral or lentiviral vectors, which represent the standard platforms for currently approved CAR-T products. Alternatively, non-viral approaches, including gene-editing technologies such as CRISPR-based systems and base-editing strategies, are increasingly being explored, particularly for the development of allogeneic CAR-T platforms currently under clinical investigation [29,30,31]. Viral vector safety is ensured by several design features, including the separation of viral genetic elements into different plasmids to prevent the generation of replication-competent viruses, the use of self-inactivating (SIN) long terminal repeats, and pseudotyping with heterologous envelope glycoproteins (e.g., GALV or VSV-G) to optimize transduction efficiency and control tropism.

#### 2.2.3. T-Cell Expansion and Release Testing

Following genetic modification, engineered T cells are expanded under controlled manufacturing conditions, including the use of bioreactors, to achieve the target therapeutic dose. The final cellular product undergoes extensive quality control and release testing, including assessment of identity, viability, potency, sterility, and absence of microbial contamination [32,33]. After approval for release, CAR-T cells are cryopreserved and transported back to the treating center for administration.

#### 2.2.4. Lymphodepleting Conditioning

Before CAR-T cell infusion, patients receive lymphodepleting chemotherapy, typically administered 3–7 days before infusion, to reduce endogenous lymphocyte competition, enhance homeostatic cytokine availability (particularly IL-7 and IL-15), promote CAR-T cell expansion and persistence, and modulate the tumor microenvironment [34]. The final CAR-T cell product is then administered intravenously as a single infusion.

#### 2.2.5. In Vivo CAR-T Cell Activity

Following infusion, CAR-T cells recognize their target antigen on malignant cells through MHC-independent antigen recognition, undergo in vivo expansion, and mediate cytotoxic tumor-cell killing through direct cellular cytotoxicity and cytokine-mediated immune activation. The vein-to-vein time, defined as the interval between leukapheresis and CAR-T cell infusion, currently ranges from approximately 3 to 5 weeks, although this interval may vary according to manufacturing platform, logistics, and institutional workflow. Recent real-world analyses have reported a median vein-to-vein time of approximately 31 days, including leukapheresis, manufacturing, release testing, and product shipment [35,36].

## 3. Post-CAR-T Relapse: Mechanism and Risk Factors

Relapse following CAR-T cell therapy remains the principal obstacle to achieving durable disease control across B-ALL, NHL, and MM. The incidence of relapse varies according to the underlying disease, CAR construct, target antigen, disease burden at infusion, and patient-related factors. Despite the remarkable complete response rates reported in pivotal clinical trials, long-term follow-up indicates that a substantial proportion of patients ultimately experience disease recurrence or progression, as reflected by the progression-free survival (PFS) outcomes reported in Table 1.

Increasing insight into the biological basis of treatment failure has revealed that post-CAR-T relapse is a multifactorial process resulting from dynamic interactions between tumor cells, CAR-T cells, and the host immune microenvironment. From a biological perspective, relapse is broadly classified into two major categories: antigen-positive relapse, in which malignant cells retain expression of the target antigen despite CAR-T failure, and antigen-negative relapse, characterized by complete or partial loss of target antigen expression, thereby enabling immune escape. This classification provides the conceptual framework for understanding the distinct mechanisms of resistance and for developing disease-specific preventive and therapeutic strategies [37,38].

### 3.1. Biological Mechanism of Relapse Post-CAR-T

**Figure 2:**
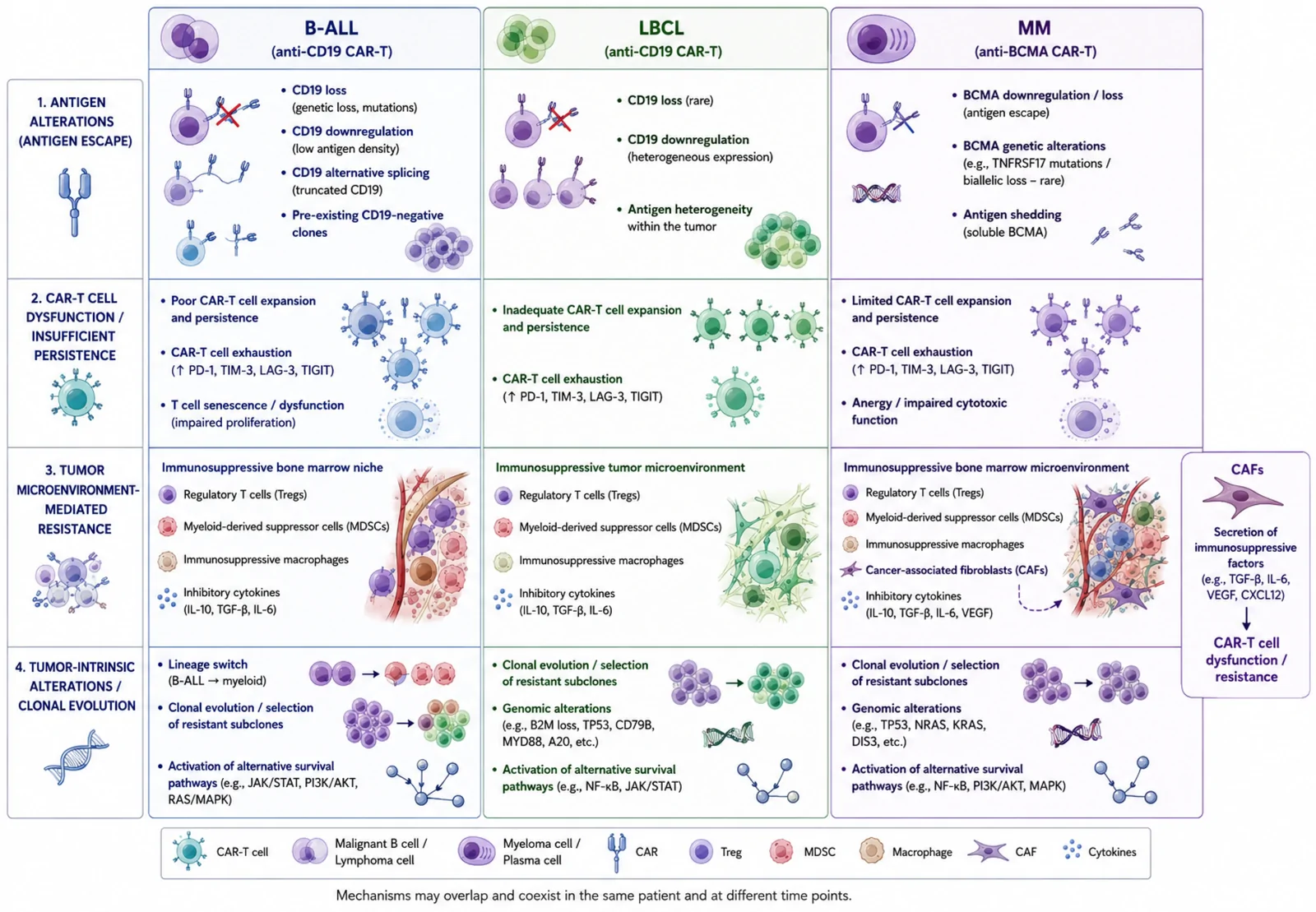
Different mechanisms of relapse after CAR-T therapy in B-ALL, LBCL, and MM.

For CD19-directed CAR-T cells, relapse can be broadly classified according to both timing (early versus late) and antigen status (CD19-positive versus CD19-negative). Although these classifications partially overlap, they reflect distinct biological mechanisms of treatment failure. Early relapse is more commonly associated with CD19-positive disease and is generally attributed to inadequate CAR-T cell expansion, functional exhaustion, or limited persistence, leading to the loss of long-term immune surveillance following recovery of normal B cells (Figure 2) [39].

In contrast, late relapse is more frequently characterized by CD19-negative disease and is primarily driven by immune escape under the selective pressure exerted by CAR-T cells. This biological classification has been most extensively characterized in B-ALL, where approximately 40–60% of post-CAR-T relapses retain CD19 expression, whereas the remaining cases exhibit complete or partial loss of CD19 [40]. By contrast, CD19-negative relapse is relatively uncommon in LBCL, in which treatment failure is more frequently associated with inadequate CAR-T cell persistence, T-cell dysfunction, and tumor-intrinsic resistance mechanisms rather than antigen escape [41]. In particular three major mechanisms of escape from CAR-T cell-mediated immunosurveillance have been described: antigen loss, immune dysfunction and T-cell exhaustion, and tumor microenvironment (TME)-mediated immunosuppression. Antigen loss, particularly CD19 loss, is well documented in B-ALL, occurring in approximately 40% of relapses, and has also been reported in approximately 33% of LBCL cases. Similar mechanisms have been described with other immunotherapeutic approaches, including CD20 loss after CD20/CD3 bispecific antibodies in follicular lymphoma and LBCL, and BCMA loss during BCMA-directed CAR-T cell therapy in MM [42,43]. Antigen escape may result from target downregulation, somatic mutations affecting antigen expression or anchoring, alternative splicing, or epigenetic alterations. A particular mechanism of antigen loss is the lineage switch. The latter occurs predominantly in KMT2A-rearranged B-ALL, whereby leukemic blasts undergo transdifferentiation toward a myeloid phenotype with concomitant loss of B-lineage antigens, thereby escaping CD19-directed CAR-T-cell recognition [44].

Immune dysfunction and CAR-T cell exhaustion represent another major mechanism of treatment failure, although their underlying mechanisms remain incompletely understood. Checkpoint-mediated inhibition by tumor cells and other components of the TME may impair CAR-T cell function, while intrinsic features of the CAR-T cell product also influence treatment durability. In MM, for example, longer responses have been associated with a higher proportion of CD8+ CAR-T cells. Finally, the TME may promote CAR-T dysfunction through immunosuppressive signaling and metabolic competition, creating conditions that impair CAR-T cell proliferation, persistence, and cytotoxic activity [39,41]. In this context, in MM, cancer-associated fibroblasts (CAFs) have been identified as key components of the tumor microenvironment (TME) [45].

### 3.2. Risk Factors for Post-CAR-T Relapse

Several clinical and biological variables have been associated with an increased risk of relapse following CAR-T therapy, although their relative contribution varies across hematologic malignancies. In B-ALL and LBCL, a high pre-infusion tumor burden consistently represents one of the strongest predictors of relapse, likely reflecting greater tumor heterogeneity, increased antigenic diversity, and enhanced selective pressure favoring immune escape [45,46]. In addition, inadequate in vivo CAR-T cell expansion and limited long-term persistence, clinically reflected by early B-cell recovery, are major determinants of CD19-positive relapse, whereas antigen loss and lineage switch predominantly account for CD19-negative relapse [40,41,42].

In MM, extramedullary disease and high-risk cytogenetic abnormalities, including del(17p), t(4;14), and t(14;16), are associated with inferior PFS following BCMA-directed CAR-T therapy [47]. Furthermore, genomic alterations involving the TNFRSF17 (BCMA) gene, particularly homozygous deletion or biallelic loss, have emerged as rare but well-characterized mechanisms of resistance by abrogating target antigen expression and recognition [43,44]. More recently, the MyCARe (Myeloma CAR-T Relapse) score, which integrates extramedullary disease, plasma cell leukemia, lenalidomide-refractory disease, and elevated serum ferritin at the time of lymphodepletion, demonstrated good discriminatory performance for predicting early relapse after BCMA-directed CAR-T therapy and may facilitate risk-adapted therapeutic strategies [47].

## 4. Strategies for Prevention and Management Post-CAR-T Relapse in B-ALL

**Figure 3:**
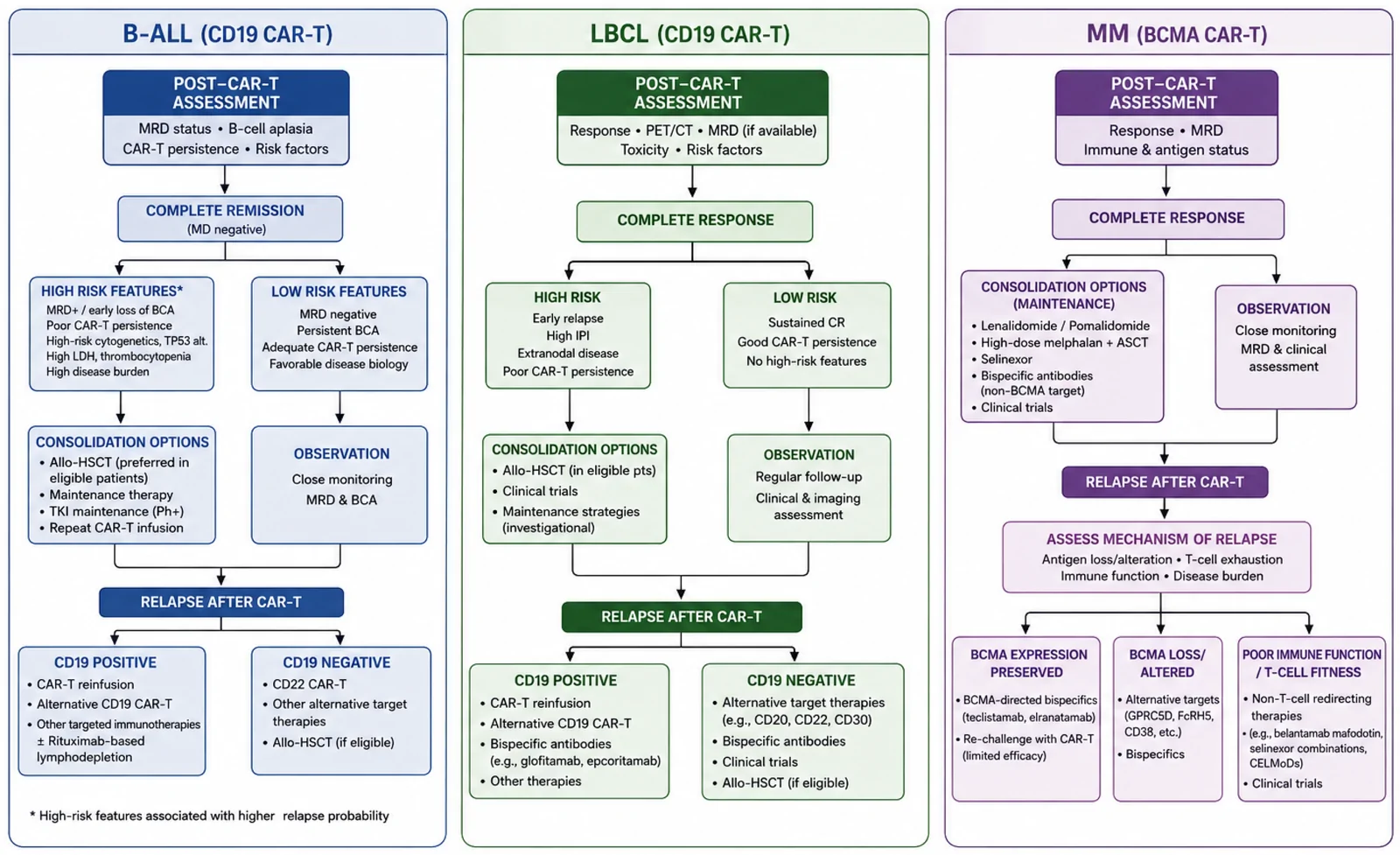
Post-CAR-T management algorithm. Abbreviations: allo-HSCT, allogeneic hematopoietic stem cell transplantation; ASCT, autologous stem cell transplantation; B-ALL, B-cell acute lymphoblastic leukemia; BCA, B-cell aplasia; BCMA, B-cell maturation antigen; CAR-T, chimeric antigen receptor T-cell therapy; CD, cluster of differentiation; CELMoDs, cereblon E3 ligase modulators; CR, complete response; GPRC5D, G protein-coupled receptor class C group 5 member D; IEC-HS, immune effector cell-associated hemophagocytic syndrome; LDH, lactate dehydrogenase; LBCL, large B-cell lymphoma; MM, multiple myeloma; MRD, measurable residual disease; OS, overall survival; PET/CT, positron emission tomography/computed tomography; Ph+, Philadelphia chromosome-positive; PFS, progression-free survival; RFS, relapse-free survival; TCEs, T-cell engagers; TKI, tyrosine kinase inhibitor; TP53, tumor protein p53.

Two main therapeutic approaches can be considered in the post-CAR-T setting in B-ALL: consolidation therapy, intended to prevent relapse in patients at high risk of treatment failure, and salvage therapy, reserved for patients who experience overt disease relapse (Figure 3).

### 4.1. Post-CAR-T Consolidation in B-ALL

The role of consolidative allo-HSCT following CD19-directed CAR-T cell therapy in adults with B-ALL remains controversial. Although relapse after CAR-T therapy occurs in approximately 40–60% of patients, the potential benefit of allo-HSCT must be carefully balanced against transplant-related mortality (TRM), morbidity, and the possibility that CAR-T cells alone may induce durable long-term disease control.

Data from prospective clinical trials suggest that a proportion of patients can maintain prolonged remission without routine consolidative transplantation. In the ELIANA trial evaluating tisa-cel in children and young adults with R/R B-ALL, only 22% of patients subsequently underwent allo-HSCT. After censoring for transplantation and additional anticancer therapies, relapse-free survival (RFS) was 58% at 24 months and 52% at 36 months, supporting the ability of CAR-T cells to induce sustained remission without mandatory transplantation [1]. Similarly, in the ZUMA-3 trial evaluating brexu-cel in adults with R/R B-ALL, only 19% of patients proceeded to allo-HSCT. Although transplantation was associated with a longer duration of response (44.2 versus 18.6 months), the impact on OS remained uncertain, suggesting that allo-HSCT may benefit selected patients rather than representing a universal post-CAR-T strategy [2].

Evidence from real-world studies remains heterogeneous. Some retrospective analyses have suggested improved outcomes with consolidative allo-HSCT, particularly among patients with high-risk features such as persistent measurable residual disease (MRD) or poor CAR-T persistence, whereas other studies have failed to demonstrate a clear survival advantage. These discrepancies are likely related to retrospective design, limited sample size, and selection bias. Consequently, the role and timing of allo-HSCT after CAR-T therapy remain areas of active investigation [48].

Current evidence supports a risk-adapted approach based on disease biology, MRD status, and CAR-T kinetics. Patients with adverse prognostic characteristics, including high-risk cytogenetic abnormalities, TP53 alterations, elevated lactate dehydrogenase (LDH), thrombocytopenia, high disease burden before infusion, persistent MRD after CAR-T infusion, early loss of B-cell aplasia (BCA), or evidence of limited CAR-T persistence, appear to have a higher probability of relapse and may represent the population most likely to benefit from consolidative transplantation [49].

MRD monitoring represents an important tool for post-CAR-T risk stratification. Next-generation sequencing (NGS)-based MRD assessment performed in prospective studies, including ELIANA and ENSIGN, demonstrated high sensitivity for predicting relapse after CAR-T therapy. Accordingly, detectable MRD during early post-infusion evaluation or loss of BCA within the first months after treatment may identify patients who should be considered for pre-emptive consolidation strategies, including allo-HSCT [50].

For patients who are not eligible for transplantation, alternative consolidation approaches have been explored, including maintenance chemotherapy, tyrosine kinase inhibitors (TKIs) in Philadelphia chromosome-positive (Ph+) ALL, and repeat CAR-T infusion. In pediatric patients, maintenance chemotherapy combining oral mercaptopurine, weekly methotrexate, periodic intrathecal therapy, and intermittent vincristine/dexamethasone has shown encouraging outcomes, with survival rates comparable to those observed after allo-HSCT in selected cohorts [51].

Among adults with Ph+ B-ALL, TKI maintenance represents a particularly attractive transplant-sparing strategy. Othman et al. reported the feasibility of post-CAR-T maintenance with TKIs, including asciminib, ponatinib, and imatinib, in patients with Ph+ B-ALL, suggesting potential for prolonged disease control [52]. More recently, Chen et al. investigated sequential anti-CD19 CAR-T therapy combined with autologous CD19-positive feeding T cells (FTCs) and TKI maintenance in Ph+ B-ALL. All evaluable patients achieved complete remission, including the majority achieving complete molecular response, with encouraging relapse-free survival and overall survival after extended follow-up, supporting this approach as a potential transplant-sparing strategy [52].

Repeat CAR-T infusion has also been evaluated, particularly in patients experiencing loss of CAR-T persistence or recovery of normal B cells. In a retrospective analysis, second infusion of CD19-directed CAR-T cells resulted in complete remission in a subset of patients, with responders achieving prolonged B-cell aplasia, suggesting restoration of CAR-T-mediated immune surveillance [53]. However, larger prospective studies are required to define the optimal timing, CAR construct, and patient selection criteria.

### 4.2. Management of Relapse of B-ALL Post-CAR-T

The prognosis of patients experiencing relapse after CD19-directed CAR-T therapy remains unfavorable; however, long-term disease control may still be achieved in selected patients when treatment is adapted according to the biological mechanism of relapse.

#### 4.2.1. CD19-Positive Relapse

For patients who retain CD19 expression at relapse, therapeutic options include CAR-T reinfusion, alternative CD19-directed CAR-T products, and other targeted immunotherapies. Reinfusion of cryopreserved CD19-directed CAR-T cells may be considered, particularly in patients with late relapse and preserved CAR-T persistence. However, repeated administration of the same CAR construct may be limited by anti-CAR immune responses and impaired expansion. Gauthier et al. reported a complete remission rate of only 21% after reinfusion of the original CD19 CAR-T product, despite administration of a higher CAR-T cell dose, highlighting the challenges associated with CAR-T retreatment [53]. To overcome immune-mediated resistance, humanized or fully human CD19 CAR constructs have been developed. In a cohort of children and young adults previously exposed to CD19 CAR-T therapy, retreatment with a humanized CD19 CAR-T product resulted in a CR rate of 79%, with high rates of MRD-negative responses and encouraging relapse-free survival among responders [54]. Similarly, Ortiz-Maldonado et al. explored retreatment with CD19 CAR-T cells following rituximab-containing lymphodepletion aimed at reducing humoral anti-CAR immune responses. Although high MRD-negative remission rates were achieved, progression-free survival remained limited, suggesting that additional strategies are required to improve durability [55].

#### 4.2.2. CD19-Negative Relapse

CD19-negative relapse represents a major mechanism of resistance after CD19-directed CAR-T therapy and requires alternative antigen targeting strategies. Among these, CD22-directed CAR-T cells have shown promising activity, particularly in patients who relapse after CD19-targeted immunotherapy. Pan et al. demonstrated that CD22 CAR-T therapy induced complete remission in 70% of heavily pretreated pediatric and adult patients with B-ALL, with improved leukemia-free survival among patients subsequently undergoing allo-HSCT, suggesting a role for transplantation as consolidation after CD22 CAR-T therapy [56]. Similarly, Shah et al. reported a complete remission rate of approximately 70% in children and young adults treated with CD22 CAR-T cells after CD19 CAR-T failure; however, responses were frequently not durable, with median relapse-free survival of approximately six months [57]. CRS and IEC-HS represented relevant toxicities requiring optimization of CAR-T dosing strategies [57]. More recently, Schultz et al. reported encouraging results using a different CD22 CAR-T platform, achieving MRD-negative remission in a substantial proportion of pediatric and adult patients, although durable disease control remained mainly associated with subsequent allo-HSCT [58].

Overall, salvage strategies after CAR-T failure, including intensive chemotherapy, blinatumomab (for CD19+), inotuzumab ozogamicin, and targeted cellular therapies, may still provide a bridge toward potentially curative consolidation. In a retrospective analysis of adults relapsing after CD19 CAR-T therapy, salvage treatments enabled a proportion of patients to proceed to allo-HCT, demonstrating that durable remission remains achievable in selected cases despite CAR-T failure [48].

## 5. Strategies for Management Post-CAR-T Relapse in LBCL

Although relapse after CAR-T cell therapy in LBCL remains associated with a dismal prognosis, the therapeutic landscape has expanded considerably over the past few years. Several novel agents demonstrated clinically meaningful activity in the post-CAR-T setting, while additional promising therapies are currently being evaluated in clinical trials [59,60]. Developing a personalized and sequential treatment strategy should therefore be considered an integral component of the management of patients with LBCL undergoing CAR-T cell therapy, both before infusion and at the time of relapse. Regardless of the therapeutic approach adopted, obtaining a repeat tumor biopsy at relapse remains a cornerstone of patient management. Histological confirmation and reassessment of tumor biology, including the evaluation of CD19 and CD20 expression, are essential to guide subsequent treatment selection and optimise therapeutic sequencing (Figure 3).

Bispecific antibodies (BsAbs) CD20 × CD3 BsAbs have rapidly become a relevant therapeutic option for patients with R/R CD20-positive B-cell lymphomas, including those who relapse after CAR T-cell therapy. Several agents, such as mosunetuzumab, epcoritamab, odronextamab, and glofitamab, have shown significant antitumor activity across different clinical settings, both as monotherapies and in combination approaches, in CAR T-cell naïve patients as well as post-CAR T-cell treatment [61,62,63,64,65,66,67,68].

In the post-CAR T-cell relapse setting, BsAbs have demonstrated clinical efficacy despite the adverse prognosis associated with this population, with reported CR rates ranging approximately from 12% to 36%, varying according to the specific disease and patient characteristics. Notably, responses achieved with these agents may be prolonged, as observed with glofitamab, where the median duration of CR exceeded 19 months in clinical studies, suggesting the possibility of sustained disease control in a subset of responding patients [63,64]. Comparable activity has been reported with epcoritamab and odronextamab, which have induced relevant responses even among heavily pretreated patients, including those previously exposed to CAR T-cell therapy, reinforcing their role as valuable salvage therapies [65].

Data from real-world cohorts have further supported the efficacy observed in prospective clinical trials. In a multicenter analysis of patients treated with anti-CD20 × CD3 BsAbs after CAR T-cell failure, an ORR of 51% was reported, with CR achieved in 36% of cases [66]. These findings are in line with the results from pivotal studies, including EPCORE NHL-1, NP30179, and ELM-2, where epcoritamab, glofitamab, and odronextamab demonstrated substantial and durable activity in heavily pretreated LBCL populations, including patients previously treated with CAR T cells [67,68]. Given their favorable balance between efficacy, durability of response, and manageable toxicity profile, these agents are increasingly considered a preferred treatment option in the post-CAR T setting when access to clinical trials is not available [65].

Treatment options in the post-CAR-T setting include targeted agents, immunomodulatory approaches, radiotherapy, and, in selected patients, allogeneic hematopoietic stem cell transplantation (allo-HSCT), particularly when access to clinical trials is not available [65].

**CD19-targeting agents.** Loss or downregulation of CD19 expression represents a well-recognized mechanism of resistance to CD19-directed immunotherapies. However, the majority of patients with R/R LBCL who relapse after CAR-T cell therapy retain CD19 expression, providing a rationale for considering CD19-directed approaches in this setting. Among the available CD19-targeted therapies, tafasitamab (tafa), an Fc-enhanced anti-CD19 monoclonal antibody, and loncastuximab tesirine (lonca), an antibody–drug conjugate targeting CD19, have demonstrated clinical activity in patients with R/R LBCL, including those previously exposed to CAR-T cell therapy [69,70].

Recent evidence has also provided support for the feasibility of sequential CD19 targeting after prior exposure to CD19-directed therapies. In a large global real-world study evaluating CAR-T therapy after tafasitamab, with or without lenalidomide, prior tafa exposure did not appear to adversely affect the efficacy or safety of subsequent CAR-T treatment. Among 338 patients, CAR-T therapy resulted in an ORR of 81.1%, including a CR rate of 58.6%, although the median follow-up was relatively short at 7.4 months [71,72,73]. These findings are particularly relevant in the context of the evolving frontline treatment landscape, in which increasing use of tafasitamab-based strategies may result in a growing number of patients previously exposed to CD19-directed therapy who subsequently become candidates for CAR-T-cell treatment [74].

Encouraging activity has also been reported with loncastuximab tesirine. Real-world studies have demonstrated ORRs ranging from approximately 50% to more than 70%, although outcomes appear to be influenced by disease burden, treatment line, and previous therapies. In particular, CR rates of approximately 34% when loncastuximab was administered in earlier salvage settings and 17% in later treatment lines suggest that its efficacy may be greater when used before extensive disease progression [75]. Overall, these findings support the continued relevance of CD19 as a therapeutic target after CAR-T failure in patients with preserved antigen expression.

**Polatuzumab vedotin.** Polatuzumab vedotin (Pola), an antibody–drug conjugate targeting CD79b, represents an established treatment option for patients with R/R LBCL. In the post-CAR-T setting, Pola-based salvage regimens have demonstrated clinically meaningful activity, with reported ORRs ranging from 44% to 72% and CR rates ranging from 14% to 33% across retrospective series and clinical studies [76]. These findings support the use of Pola-based combinations as a potential salvage strategy for patients with limited therapeutic alternatives after CAR-T failure. However, despite encouraging initial response rates, the durability of disease control remains limited, with a substantial proportion of patients experiencing relatively early disease progression [76].

**Radiotherapy.** Salvage radiotherapy may provide effective local disease control and symptomatic relief in selected patients, particularly those presenting with localized or oligoprogressive relapse. Real-world studies have reported ORRs exceeding 40% in this setting [76]. Radiotherapy may therefore represent a useful component of an individualized salvage strategy, either as definitive treatment for localized disease or as part of a multimodal approach.

**Immunomodulatory agents.** Lenalidomide has emerged as a potential therapeutic option after CAR-T failure because of its broad immunomodulatory properties and ability to enhance antitumor immune responses. Preclinical studies indicate that lenalidomide can increase CD8^+^ T-cell activation, reduce regulatory T-cell-mediated immunosuppression, and promote more effective immune synapse formation. Experimental models have also suggested that lenalidomide may partially overcome T-cell exhaustion and enhance CAR-T-cell functional activity, persistence, and cytotoxic capacity, providing a biological rationale for its investigation in the post-CAR-T setting [77].

Although clinical evidence remains limited and is derived predominantly from retrospective real-world cohorts, lenalidomide-based strategies have shown encouraging activity in patients experiencing relapse after CAR-T-cell therapy. Multicenter analyses of lenalidomide-containing regimens administered as early salvage treatment after CAR-T failure reported ORRs of up to 58%, with CR rates approaching 29% [60,61,62]. These findings suggest that immunomodulatory approaches may represent a therapeutic option for selected patients, particularly when introduced early after CAR-T relapse. However, prospective studies are needed to define their optimal role and sequencing within the expanding post-CAR-T treatment landscape.

**Immune checkpoint inhibitors.** Immune checkpoint inhibitors (ICIs) have been investigated as a salvage strategy for patients with lymphoma progressing after CAR-T-cell therapy, based on the rationale that CAR-T-cell failure may be associated with T-cell exhaustion and an immunosuppressive tumor microenvironment. Available clinical evidence, derived mainly from retrospective multicenter analyses, suggests some activity in this setting, although responses are heterogeneous and generally less consistent than those reported with some other emerging post-CAR-T approaches. Across three retrospective studies, ICIs achieved ORRs ranging from 19% to 46%, with CR rates between 10% and 25% [78,79].

The likelihood of response appears to be influenced by lymphoma biology and patient selection. Among LBCL subtypes, primary mediastinal B-cell lymphoma (PMBCL) may represent a particularly relevant population for checkpoint blockade because of its characteristic genomic landscape, including frequent PD-L1 overexpression and recurrent alterations involving the PD-1/PD-L1 pathway. These biological features provide a rationale for increased sensitivity to immune checkpoint inhibition and support further investigation of ICIs in this subgroup [79,80]. However, current evidence remains limited by the retrospective nature of available studies and the absence of prospective comparative trials. Accordingly, ICIs should currently be considered primarily in selected patients, particularly those with PMBCL, preferably within clinical trials when available.

**BTK inhibitors.** The role of Bruton tyrosine kinase inhibitors (BTKis) in patients with R/R LBCL after CAR-T-cell therapy remains poorly defined, with available evidence largely limited to small retrospective series. As single-agent salvage therapy after CAR-T failure, BTKis have shown modest activity, with reported CR rates ranging from 3.8% to 18%, suggesting limited efficacy in this highly refractory population [60,61,62]. More promising results have emerged from combination strategies incorporating BTK inhibition with immunomodulatory or antibody-based agents. Notably, the combination of a BTKi with rituximab and lenalidomide has demonstrated enhanced antitumor activity in patients with R/R LBCL, achieving CR rates of approximately 30% [81]. However, the applicability of these findings to the post-CAR-T setting remains uncertain because patients previously exposed to CAR-T therapy were not included in this study. Therefore, although BTKi-based combinations represent a biologically attractive approach, their efficacy and optimal positioning after CAR-T failure require validation in prospective studies.

**Selinexor.** Selinexor, a selective inhibitor of nuclear export (SINE) targeting exportin 1 (XPO1), represents a treatment option for patients with R/R LBCL who have received at least two previous lines of systemic therapy. Clinical studies have demonstrated that selinexor can induce responses in a subset of heavily pretreated patients, although overall activity is limited and response durability is variable. Its clinical use may also be challenging because of its toxicity profile, which commonly includes hematologic adverse events, fatigue, anorexia, and other constitutional symptoms. Appropriate patient selection, proactive supportive care, and close monitoring are therefore important to optimize treatment feasibility and preserve quality of life during therapy [82].

**Allogeneic hematopoietic stem cell transplantation.** Allo-HSCT remains one of the few potentially curative strategies for selected patients who achieve disease control after salvage therapy following CAR-T-cell failure. However, the decision to proceed with transplantation requires careful patient selection and individualized assessment, given the substantial risk of transplant-related complications, particularly non-relapse mortality (NRM). Retrospective studies have reported encouraging long-term outcomes among patients undergoing allo-HSCT after achieving a response to salvage treatment, with approximately 2-year OS and PFS rates of 53% and 43%, respectively, supporting the feasibility and potential curative role of allo-HSCT in this setting [83,84].

Despite these outcomes, NRM remains a major limitation to the broader application of allo-HSCT after CAR-T relapse. Important contributors to transplant-related mortality include graft-versus-host disease (GVHD), severe infections, and transplant-related organ toxicities. Nevertheless, available retrospective evidence suggests that, in highly selected patients who achieve adequate disease control following salvage therapy and retain sufficient performance status and organ function, allo-HSCT may provide prolonged disease control and remains an important curative-intent strategy after CAR-T failure [83,84].

## 6. Strategies for Prevention and Management Post-CAR-T Relapse in MM

In MM, several antigens have been investigated as potential CAR-T targets in MM, including CD19, CD38, CD138, kappa light chain, SLAMF7, NKG2D, and GPRC5D; however, BCMA emerged as the first and most extensively studied target in this disease [85]. Ide-cel and Cilta-cel, both directed against BCMA, were the first CAR-T therapies approved for heavily pre-treated MM patients, most of whom were triple-class exposed and refractory (previously exposed to lenalidomide, bortezomib and daratumumab), with median PFS of 8.8 months for Ide-cel and 35.9 months for Cilta-cel [8,9,10]. These products have since received approval for earlier lines of treatment, with encouraging outcomes reported in less heavily pre-treated populations [86]. Despite these advances, achieving a plateau in survival curves remains challenging, reflecting two intrinsic features of MM biology. The first is clonal heterogeneity, which favours the emergence of antigen-negative or antigen-low subclones capable of escaping CAR-T-mediated immune pressure. The second is the highly deregulated bone marrow microenvironment, which sustains an immunosuppressive niche that limits CAR-T expansion, persistence, and cytotoxic function. New CAR-T products targeting alternative antigens or incorporating modified constructs are being developed to address these limitations; nevertheless, a durable plateau in survival has yet to be consistently achieved. This challenge underscores the critical role of consolidation strategies following CAR-T infusion in MM, aimed at counteracting antigen escape, reinforcing the immune response, and converting deep but transient remissions into durable ones (Figure 3) [87].

### 6.1. Consolidation Post-CAR-T

Preliminary data on post-CAR-T maintenance therapy remain limited to small, single-arm studies. In a Chinese cohort of 39 relapsed MM patients, high-dose melphalan followed by autologous stem cell transplantation after BCMA-directed CAR-T improved both PFS (*p* = 0.026) and OS (*p* = 0.04) compared with lenalidomide ± proteasome inhibitors [88]. Selinexor, administered both before and after BCMA-CAR-T infusion, showed early promise by upregulating BCMA expression and enhancing CAR-T activity in two treated patients [89]. Immunomodulatory agents have also been explored in this context: lenalidomide proved tolerable and capable of sustaining MRD negativity for over two years in high-risk, newly diagnosed MM patients receiving sequential anti-CD19/BCMA CAR-T, while maintenance with lenalidomide or pomalidomide has been associated with late CAR-T reactivation and improved responses in both newly diagnosed and relapsed/refractory MM populations [89]. Although encouraging, these findings remain preliminary, are drawn from uncontrolled studies, and require validation in larger, randomised settings. Beyond immunomodulatory approaches, other T-cell-redirecting therapies—particularly bispecific antibodies directed against a target distinct from BCMA—represent an appealing consolidation strategy following BCMA-CAR-T [90].

### 6.2. Management of Relapse in MM Post-CAR-T

Despite the availability of multiple novel therapeutic strategies, outcomes after CAR-T relapse remain unsatisfactory in real-world practice, with reported median PFS of 3.5 months and median OS of 17.9 months, highlighting the urgent need for more effective treatment approaches and rational sequencing of subsequent therapies [91]. The management of relapse after CAR-T cell therapy therefore represents a major clinical challenge, particularly regarding the optimal selection and timing of subsequent immunotherapeutic interventions. Treatment decisions require integration of multiple host- and disease-related factors that may influence response to further immune-based strategies [91].

Among host-related determinants, the functional status of the immune system, and particularly the degree of T-cell exhaustion within the tumor microenvironment, may significantly affect the activity of T-cell-redirecting therapies and other immune-based approaches [92]. Tumor-related characteristics are equally relevant when considering sequential antigen-directed therapies. Mechanisms of antigen escape, including loss or alteration of target expression, may compromise the efficacy of repeated targeting of the same antigen. Even before exposure to BCMA-directed therapies, monoallelic loss of TNFRSF17, the gene encoding BCMA located on chromosome 16p, can be detected in approximately 5–10% of patients. Following treatment pressure, additional resistance mechanisms, including biallelic BCMA loss or mutations involving the extracellular domain of BCMA, have been described in approximately 40% of patients relapsing after BCMA-targeted therapies [92].

In this context, incorporation of biomarkers assessing both immune competence and antigen integrity, including NGS-based approaches and RNA sequencing, may represent an important step toward personalized treatment algorithms and rational sequencing of immunotherapies. Re-challenge with the same CAR-T construct has shown limited efficacy after disease relapse, as reported in both the KarMMa and CARTITUDE-1 studies, suggesting that repeated targeting of the same antigen may be compromised by mechanisms of resistance, including antigen escape and impaired immune function [93]. The activity of BCMA-directed T-cell engagers (TCEs) following BCMA-CAR-T failure has been variable. In cohort C of the MajesTEC-1 study, 15 patients previously treated with BCMA-directed CAR-T cells and subsequently exposed to teclistamab achieved an ORR of 53%, with a median PFS of 4.4 months [94]. Similarly, a pooled analysis evaluating elranatamab after prior BCMA-directed CAR-T therapy reported an ORR of 53%, with a longer median PFS of 10 months [94]. These results highlight the importance of considering both antigen availability and residual T-cell functional capacity when selecting patients for sequential T-cell-redirecting therapies.

Given the possibility of BCMA loss or genetic alterations affecting target expression, redirecting treatment toward alternative antigens represents an attractive therapeutic strategy. Among emerging targets, GPRC5D-directed bispecific antibodies have demonstrated particularly encouraging activity after BCMA-directed therapies. In the MonumenTAL-1 study, 56 patients previously exposed to BCMA-targeted CAR-T therapy received talquetamab, achieving an ORR of 71% and a median duration of response of 12.3 months, representing one of the most favorable outcomes reported to date in the post-BCMA CAR-T setting [95].

In selected patients, therapeutic approaches that do not rely on T-cell redirection may be advantageous, particularly when profound immune dysfunction or impaired T-cell fitness limits the efficacy of further immunotherapies. Belantamab mafodotin, a BCMA-directed antibody–drug conjugate, has demonstrated activity in patients previously treated with BCMA-targeted therapies [96]. Similarly, combination strategies incorporating selinexor have shown encouraging efficacy, with the STOMP trial reporting an ORR of 64% in heavily pretreated patients [97]. In addition, next-generation immunomodulatory agents such as CELMoDs (cereblon E3 ligase modulators) are emerging as potential therapeutic options. In the CC-220-MM001 trial, iberdomide administered to 41 patients previously exposed to BCMA-directed therapies resulted in an ORR of 34%, with a median duration of response of 7.5 months [98].

## 7. Future Prospects for CAR-T Cell Therapy

CAR T-cell therapy has fundamentally changed the treatment paradigm of B-cell malignancies, providing unprecedented rates of response and durable disease control in a subset of patients with high-risk B-ALL, LBCL, and MM [1,3,8]. Nevertheless, relapse remains the major limitation of this approach and reflects the complex interplay between disease biology, antigen expression, tumor burden, and the quality and persistence of the CAR T-cell product [19,21,37]. Importantly, the clinical meaning of post-CAR T relapse has evolved. Rather than representing an invariably fatal event, relapse should increasingly be regarded as a biologically heterogeneous condition in which the underlying mechanism, timing, and disease kinetics may identify opportunities for effective subsequent therapy.

A major lesson emerging across B-cell malignancies is that CAR T-cell failure is not a single biological entity. Relapse may result from antigen loss or downregulation, inadequate CAR T-cell expansion or persistence, T-cell dysfunction, an immunosuppressive tumor microenvironment, or the selection of resistant tumor clones. The relative contribution of these mechanisms differs across diseases and individual patients, explaining the marked heterogeneity of outcomes after CAR T-cell therapy. This observation has important clinical implications: therapeutic decisions should not rely solely on the fact that relapse has occurred, but should incorporate antigen status, disease kinetics, molecular characteristics, previous treatment exposure, immune fitness, and the timing of relapse. Thus, post-CAR T management is progressively moving from a uniform salvage approach toward a mechanism-driven and patient-specific strategy.

This concept also highlights the importance of risk assessment across the entire CAR T-cell treatment pathway. Pre-infusion disease burden, molecular risk, T-cell fitness, and other patient- and disease-related characteristics may influence the probability of durable disease control, while early assessment of CAR T-cell expansion, persistence, MRD, and disease kinetics may provide dynamic information on treatment effectiveness [39,40]. Rather than using these parameters independently, their integration could enable longitudinal risk stratification and identify patients who may benefit from consolidation or early intervention before overt clinical relapse. In this context, MRD assessment, molecular profiling, and immune monitoring should be considered complementary tools for defining the depth and durability of response.

The implications of this approach differ according to disease context. In B-ALL, the central clinical question is increasingly which patients require consolidation after achieving remission with CAR T-cell therapy and which can safely remain under observation. Allo-HSCT remains a potentially curative strategy for selected high-risk patients, particularly those with persistent MRD, adverse cytogenetic or molecular features, or evidence of limited CAR T-cell persistence [49,54]. However, exposing all responders to transplantation would result in unnecessary toxicity in patients who may already have durable CAR T-mediated disease control. Dynamic post-infusion assessment may therefore be particularly valuable in B-ALL to distinguish patients who require additional therapy from those who may be successfully monitored without immediate consolidation.

In LBCL, the major shift has instead been the rapid expansion of effective therapies available after CAR T-cell failure. The emergence of BsAbs, antibody–drug conjugates, and other targeted approaches has demonstrated that meaningful responses can still be achieved after CAR T-cell therapy [64,65,66]. Consequently, the key challenge is no longer simply identifying a salvage treatment, but determining which treatment should be used, and when, according to relapse kinetics, antigen expression, previous exposure, and patient fitness. This evolving therapeutic landscape also raises an important broader question regarding treatment sequencing, as the increasing efficacy of frontline therapies may modify the population ultimately referred for CAR T-cell therapy and potentially influence its optimal positioning within treatment algorithms.

In MM, where most currently approved CAR T-cell approaches target BCMA, the experience after relapse further illustrates the importance of antigen and pathway diversification. Resistance may involve BCMA loss or downregulation, clonal selection, or impaired T-cell function, supporting the rationale for switching the therapeutic target rather than simply repeating the same biological strategy. Accordingly, targeting alternative antigens such as GPRC5D, together with BsAbs and other emerging immune-based approaches, provides a framework for sequential treatment after BCMA-directed CAR T-cell failure [86,87,88]. The MM experience therefore reinforces a general principle applicable across diseases: when relapse reflects target-specific resistance, therapeutic sequencing should incorporate biological diversification rather than relying exclusively on intensification of the same treatment strategy.

Taken together, these observations suggest that the future of CAR T-cell therapy will depend as much on how patients are selected and treated over time as on how the cellular products themselves are engineered. Next-generation CAR platforms may address some of the biological limitations responsible for treatment failure by improving tumor specificity, overcoming antigen heterogeneity, and increasing resistance to immunosuppressive environments. Synthetic biology approaches, including logic-gated CARs such as AND- and OR-gate circuits, may enable more selective recognition of malignant cells, while armored CARs and other engineered constructs may modify the tumor microenvironment [99,100,101]. Similarly, gene-editing strategies aimed at reducing T-cell exhaustion or enhancing persistence, including approaches targeting regulatory pathways such as TET2, may improve CAR T-cell fitness and durability.

Ultimately, the most effective strategy for preventing and treating relapse is unlikely to rely on a single intervention. Rather, durable disease control will require an integrated approach combining appropriate patient selection, optimization of CAR T-cell fitness, early biological monitoring, mechanism-based detection of resistance, and rational sequencing of subsequent therapies. The major unmet need is therefore to translate these biological insights into clinically validated algorithms that can identify, at predefined time points, which patients are likely to achieve durable remission with CAR T-cell therapy alone, which require consolidation, and which may benefit from early salvage intervention. Such a dynamic, personalized framework may represent the next major step in moving CAR T-cell therapy from a highly effective treatment for selected patients toward a more predictable and durable therapeutic strategy.

## 8. Conclusions

CAR T-cell therapy has profoundly transformed the prognosis of selected patients with high-risk B-ALL, LBCL, and MM, establishing cellular immunotherapy as a cornerstone of treatment in these diseases. However, the therapeutic algorithms for these malignancies are undergoing rapid evolution as an expanding armamentarium of approved agents and investigational immunotherapies becomes available. This progress is expected to reshape current treatment paradigms, moving from isolated therapeutic interventions toward dynamic, sequential, and biology-driven strategies. Future treatment decisions will increasingly rely on individualized therapeutic sequencing based on disease biology, mechanisms of resistance, prior therapies, and patient-specific characteristics. The integration of CAR T-cell therapy with BsAbs, antibody–drug conjugates, novel cellular therapies, and emerging targeted agents will further refine patient management. Ultimately, continued technological advances in CAR engineering are expected to overcome current limitations of cellular therapies [102].

## Figures and Tables

**Table 1:** Approved CAR T-cell products for B-cell malignancies: main clinical trials, efficacy, safety and long-term outcomes.

CAR-T Product	Target	Co-Stimulatory Domain	Approved Indication	Study Design	Treatment Setting	Prior CAR-T Exposure	N	Best Response (ORR/CR)	Median PFS (Months)	Median OS (Months)	Grade ≥3 CRS (%)	Grade ≥3 ICANS (%)	Longest Follow-Up	Evidence Level	Ref
Tisagenlecleucel (Kymriah)	CD19	4-1BB	Pediatric/AYA R/R B-ALL	Phase 2, single-arm	2L+/≥2nd relapse; or 1st relapse refractory to reinduction	No (excluded)	75	ORR 82%; CR/CRi 81%	NR	NR	47	13	61 months	Phase 2	[1]
Tisagenlecleucel (Kymriah)	CD19	4-1BB	R/R LBCL	Phase 2, single-arm	R/R after ≥2 lines; ASCT-ineligible, declined, or post-ASCT progression	No (excluded)	115	ORR 52%; CR 40%	2.9	11.1	23	12	40 months	Phase 2	[2]
Tisagenlecleucel (Kymriah)	CD19	4-1BB	R/R Follicular lymphoma	Phase 2, single-arm	≥2 prior lines or relapse after autologous SCT	No (excluded)	97	ORR 86%; CR 69%	NR	NR	0	3	29 months	Phase 2	[5]
Axicabtagene ciloleucel (Yescarta)	CD19	CD28	R/R LBCL	Phase 1/2, single-arm	R/R/refractory after ≥2 prior lines; primary refractory or early relapse	No (excluded)	111	ORR 83%; CR 58%	5.9	25.8	11	32	63 months	Phase 1/2	[3]
Axicabtagene ciloleucel (Yescarta)	CD19	CD28	Second-line LBCL	Phase 3, randomized	2L LBCL with primary refractory disease or relapse ≤12 months after first-line therapy	No	359	ORR 83%; CR 65%	14.7	NR	6	21	47 months	Phase 3 RCT	[4]
Brexucabtagene autoleucel (Tecartus)	CD19	CD28	Adult R/R B-ALL	Phase 1/2, single-arm	R/R adult B-ALL after prior therapies	No (excluded)	71	CR/CRi 71%	11.6	25.4	24	25	39 months	Phase 1/2	[2]
Brexucabtagene autoleucel (Tecartus)	CD19	CD28	R/R Mantle cell lymphoma	Phase 2, single-arm	R/R after chemoimmunotherapy and BTK inhibitor	No (excluded)	74	ORR 91%; CR 68%	25.8	46.6	15	31	35 months	Phase 2	[6]
Lisocabtagene maraleucel (Breyanzi)	CD19	4-1BB	R/R LBCL	Phase 1, single-arm	R/R after ≥2 prior lines	No (excluded)	269	ORR 73%; CR 53%	6.8	27.3	2	10	36 months	Phase 1	[7]
Idecabtagene vicleucel (Abecma)	BCMA	4-1BB	R/R Multiple myeloma	Phase 2, single-arm	≥3 prior lines; exposed/refractory to PI, IMiD and anti-CD38 therapy	No (excluded)	128	ORR 73%; CR/sCR 33%	8.8	24.8	5	3	38 months	Phase 2	[8]
Ciltacabtagene autoleucel (Carvykti)	BCMA	4-1BB	R/R Multiple myeloma	Phase 1b/2, single-arm	≥3 prior lines or double-refractory; PI, IMiD and anti-CD38 exposed	No	97	ORR 98%; sCR 82%	34.9	NR	4	10	38 months	Phase 1b/2	[10]
Obecabtagene autoleucel (Aucatzyl)	CD19	4-1BB (Fast-Off CAR)	Adult R/R B-ALL	Phase 1b/2, single-arm	R/R adult B-ALL after prior therapies	No	153	CR/CRi 77%	NR	NR	3	7	21 months	Phase 1b/2	[11]

Abbreviations: AYA, adolescents and young adults; ASCT, autologous stem-cell transplantation; BCMA, B-cell maturation antigen; CAR, chimeric antigen receptor; CR, complete response; CRi, complete remission with incomplete hematologic recovery; CRS, cytokine release syndrome; ICANS, immune effector cell-associated neurotoxicity syndrome; LBCL, large B-cell lymphoma; MM, multiple myeloma; NR, not reached/not reported; ORR, overall response rate; OS, overall survival; PFS, progression-free survival; RCT, randomized controlled trial; sCR, stringent complete response.

## Data Availability

No new data were created or analyzed in this study. Data sharing is not applicable to this article.
